# Overweight among Medical Students in a Tertiary Care Center: A Descriptive Cross-sectional Study

**DOI:** 10.31729/jnma.6607

**Published:** 2021-08-31

**Authors:** Chandra Kala Rai, Sarbada Makaju

**Affiliations:** 1 Department of Physiology, Kathmandu Medical College, Duwakot, Bhaktapur, Nepal; 2 Department of Anatomy, Kathmandu Medical College, Duwakot, Bhaktapur, Nepal

**Keywords:** *anthropometry*, *body mass index*, *overweight*, *prevalence*

## Abstract

**Introduction:**

Today's generation is facing an increased prevalence of overweight and obesity. It may be genetic or habitual due to overeating of junk foods and sedentary lifestyle. It directly affects an individual personality and health. The main aim of this study is to find out the prevalence of overweight among medical students in a tertiary care center.

**Methods:**

This is a descriptive cross-sectional study done in 385 students in a tertiary care hospital from 25th January 2020 to 28th February 2021. The sample was collected by simple random sampling method after the approval from the Institutional Review Committee of Kathmandu Medical College and Teaching Hospital. Height in meter and weight in kilogram of students were measured to calculate body mass index. Data were analyzed by using Statistical Package of Social Science software version 16. Point estimate at 95% confidence interval was calculated along with frequency and proportion for binary data.

**Results:**

Among 385 students, 75 (19.48%) (95% Confidence Interval= 15.53%-23.44%) were overweight. Fifty-seven (14.85%) males and 18 (4.69%) females were overweight respectively. In total, there were 197 (51.01%) males and 188 (48.99%) females.

**Conclusions:**

The current study shows the prevalence of overweight among medical students is slightly higher than in studies done in similar settings.

## INTRODUCTION

Overweight is the most common health problem leading to a marked increase in mortality and morbidity in the entire world. The body condition with excessive or abnormal fat accumulation is overweight and obesity.^1^ A person is considered overweight if his/her body mass index (BMI) is 25-29.9, and obese BMI is over 30.^2^ Based on the revised guidelines for the Asian population, individuals are classified as overweight if their BMI is 23-24.9, pre-obese ≥25-29.9 and obese ≥ 30.^3^

Overweight is not just aesthetic consideration. The metabolic changes can lead to serious health problems and increase risk of diseases like diabetes mellitus, hypertension, orthopedic complications, psychological disorders etc.^4^ Nowadays, people with any age group, gender, ethnicity and socio-economic background are equally affected.^5^ But, there are insufficient data of overweight among Nepalese medical students.

Overweight and obesity are common physical problems. So, the objective of this study is to find out the prevalence of overweight among medical students in a tertiary care center.

## METHODS

A descriptive cross-sectional study was carried out among undergraduate healthy medical students, aged between 17 to 25 years, in the Department of Physiology of Kathmandu

Medical College and Teaching Hospital. The study was started from 25^th^ Jan 2020 to 28^th^ Feb 2021 with approval of the Institutional Review Committee (IRC) of Kathmandu Medical College and Teaching Hospital (reference number: 130120204). The sample was selected by a simple random sampling method. The sample size was calculated by using the following formula,

n = Z^2^ × p × q / e^2^

  = (1.96)^2^ × 0.5 × 0.5 / (0.05)^2^

  = 385

where,

n = sample size,
Z = 1.96 for 95% Confidence Interval (CI),
p = prevalence of overweight for maximum sample size, 50%
q = 1-p
e = margin of error, 5%

Based on the above formula, the minimum sample size was 385.

Height in meter and weight in kilogram (kg) of students were measured to calculate Body Mass Index (BMI) by Prestige height and weight measuring scale (Stadiometer). The formula, weight in kg divided by height in meter square was used to calculate the BMI and unit is kg/m^2^. According to WHO (World Health Organization), "Asian Criteria" for BMI cut off point 23-24.99 is overweight, pre-obese ≥25-29.9 and obese ≥ 30. This "Asian Criteria" is used to analyze overweight and obesity in this study.

Data was analyzed by using Statistical Package of Social Science (SPSS) software version 16. Point estimate at 95% CI was calculated along with frequency and proportion and subgroup analysis was done on the basis of gender.

## RESULTS

**Figure 1:**
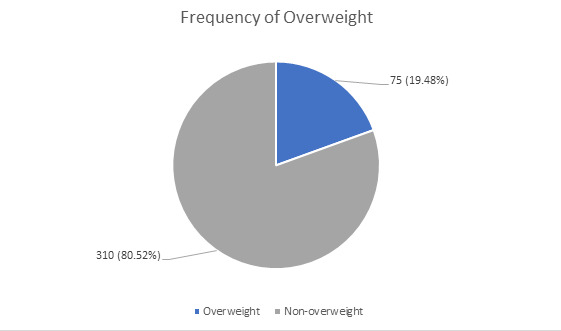
Frequency of overweight among total population students (n= 385).

In the present study, total 385 students, 75 (19.48%) (95% Confidence Interval = 15.53% - 23.44%) were overweight and mean age was 19.95±1.18 (Figure 1).

In total, overweight males were 57 (14.81%) and females were 18 (4.67%) respectively. In total, male 197 (51.01%) and female 188 (48.99%) were assessed for BMI for being overweight (Table 1).

**Table 1:** Prevalence of overweight in different gender.

Gender	Overweight n (%)	Total n (%)
Male	57 (14.81)	197 (51.02)
Female	18 (4.67)	188 (48.83)
Total	75 (19.48)	385 (100)

## DISCUSSION

In our findings, the prevalence of overweight was slightly higher than a study done by Kishore S, the study showed 14.6% medical students were overweight.^6^ Another study by Deotale M. had much less prevalence of overweight (9.3%) in medical students.^7^ A study by Rui Wang showed higher range of overweight (32.3%) than present study which was done in adult population. But the gender specific result was similar higher among male (34.3%) than female (30.2%).^8^ Kumar A. study showed contrast result of overweight in fifth batch male students, low (1%) than female students (4.9%) but similar finding was there in IV^th^ batch students, overweight male (12.15%) and females were (4.9%).^9^ Similar result was reported by Thomas E 39.8% overweight in boys and 25.5% in girls. Male showed more prevalence with overweight than girls.^10^

In contrast to the current study, the prevalence of overweight is higher in females by Gopalkrishnan 15.7% and 13.7% males.^1^ Magda Antal 19.6% in girls and 18.1% boys.^11^

A study by Gabriel L has similar result of overweight 20.26%.^12^ Rabanales-Sotos J study showed a bit higher overweight 26.5% than current study^13^ and Khan ZN showed 10.2% overweight students.^14^

This study was conducted only in basic science medical students of an institute Kathmandu Medical College, there were some limitations of the study. The study is confined only to medical students with similar age group and socio-economic backgrounds, so, the result cannot be generalized in population. The larger sample size, wider range of age and socio-economic, education background might have greater value in research.

## CONCLUSIONS

The current study shows the prevalence of overweight students is a bit higher than other similar studies in students. The male students show a higher rate of overweight than female students. It suggests that the prevalence of overweight is gender specific. But a person's age factors, stress level, day to day habits like exercise and eating can influence it. Overweight can lead to serious health problems and can increase the risk of many diseases like diabetes mellitus, hypertension, psychological disorders and many more. If the students can be counseled about the importance of normal BMI and healthy habits, many serious health issues can be avoided.
